# A Thermoset Shape Memory Polymer-Based Syntactic Foam with Flame Retardancy and 3D Printability

**DOI:** 10.1021/acsapm.1c01596

**Published:** 2022-01-26

**Authors:** Rubaiyet Abedin, Xiaming Feng, John Pojman, Samuel Ibekwe, Patrick Mensah, Isiah Warner, Guoqiang Li

**Affiliations:** † Department of Mechanical Engineering, Southern University and A&M College, Baton Rouge, Louisiana 70813, United States; ‡ Department of Mechanical & Industrial Engineering, Louisiana State University, Baton Rouge, Louisiana 70803, United States; § Department of Chemistry, Louisiana State University, Baton Rouge, Louisiana 70803, United States

**Keywords:** shape memory polymer, syntactic foam, flame retardancy, stress recovery, 3D printing

## Abstract

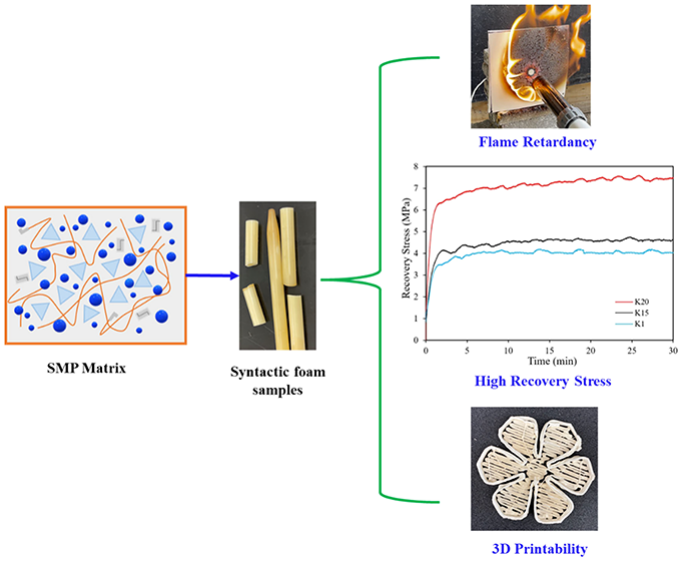

Here we report a thermoset shape memory polymer-based syntactic foam inherently integrated with flame retardancy, good mechanical properties, excellent shape memory effect, and 3D printability. The syntactic foam is fabricated by incorporating a high-temperature shape memory polymer (HTSMP) as the matrix, with 40 vol % hollow glass microspheres (HGM) K20, K15, and K1 as fillers. Compressive behavior, strain-controlled programming followed by free recovery, stress recovery, and flame retardancy of these three syntactic foams were studied. Dynamic mechanical analysis and thermal characterization validate their high glass transition temperature (*T*_g_ = ∼250 °C) and excellent thermal stability. Our results suggest that the foam consisting of K20 HGM exhibits high compressive strength (81.8 MPa), high recovery stress (6.8 MPa), and excellent flame retardancy. Furthermore, this syntactic foam was used for three-dimensional (3D) printing by an extruder developed in our lab. Honeycomb, sinusoidal shapes, and free-standing helical spring were printed for demonstration. This high-temperature photopolymer-based syntactic foam integrated with high *T*_g_, flame retardancy, high recovery stress, and 3D printability can be beneficial in different sectors such as aerospace, construction, oil and gas, automotive, and electronic industries.

## 1 Introduction

Polymeric foams have attracted a lot of attention since they were first developed in 1931^1^ because of the high demands for certain material properties such as ductility, lightweight, insulation, softness, sound and shock absorption capabilities, and so on. Solid polymeric foams can be either open-cell foam, close-cell foam, or a combination of both. When used as a sandwich core material, open-cell foams result in low flatwise compressive strength and modulus in sandwich structures,^2^ and because of water absorbency, its applications become limited in a high moisture environment including underwater structures as well as outdoor constructions such as sealant for expansion joint in concrete pavement or bridge deck.^3,4^ Compared to open-cell foams, close-cell foams or syntactic foams are studied intensively over the past six decades because of their high stiffness, low moisture absorption, excellent compressive and hydrostatic strength, and dimensional ability.^5^ Syntactic foams are polymer composite materials filled with hollow spherical particles such as glass microballoons or microspheres, metallic microspheres, polymeric microspheres, and ceramic microspheres.^6^ The presence of the hollow space of the microspheres lowers the density of the foam whereas the rigid wall material enhances the stiffness, resulting in high specific properties of the foam. Syntactic foam has gained popularity for its enhanced mechanical performance and insulating capability and is widely used in different sectors such as marine, automotive, sports, aerospace, deep-sea buoyancy materials, coating, electromagnetic shielding, and so on.^7^ Syntactic foams are often used as core materials in sandwich composites to provide enhanced bending stiffness and desired compressive strength of the sandwich structures.^8^ In the past three decades studies have been conducted to investigate the structure–property correlations for syntactic foams and mechanisms for tailoring different properties such as mechanical, electrical, and thermal properties.^9,10^ Most widely used syntactic foams are embedded with glass microballoons in different thermosetting polymers such as epoxy,^11^ polyurethane,^12^ vinyl,^13^ phenolic polymers,^14^ and so on. The mechanical properties of syntactic foams mostly depend on the microstructure. Many studies reported that the properties of the syntactic foam can be tuned by using microspheres of different mean diameters and wall thicknesses in varying volume fractions in the matrix materials.^15^ Li and Jones have presented a detailed study of the effect of microstructure on the mechanical properties of syntactic foams.^3^

Recent development in syntactic foam includes incorporating shape memory polymer (SMP) matrix as the binder of the syntactic foam.^16,17^ Shape memory polymers can keep a temporary shape and regain their original shape under different external stimuli such as light, heat, magnetic fields, moisture, and electricity.^18,19^ Incorporating SMP as the polymer matrix in syntactic foam introduces the shape memory functionality and improves mechanical properties of the foam, achieves watertightness, and provides damage healing capability.^20−24^ The mechanical properties and durability of thermoset SMP based syntactic foams were also investigated.^25−27^ This type of SMP-based foam can be used in load-bearing structural applications, deployable structures, and so on. In addition to the one-way shape memory polymer-based syntactic foam, Lu et al. have investigated the reversible bidirectional actuation behavior of a two-way SMP-based syntactic foam which enables applications in different fields such as soft robots, biomedical devices, sealants, and so on.^28^ Prima et al. have studied the thermomechanical storage and recovery behavior of thermoset SMP foams under different deformation conditions.^29^ Moreover, when the programmed shape of SMPs is partially or fully confined during recovery, it can generate a force. This recovery force can do positive work on the surroundings and can be useful in some heavy-duty engineering structures.^30^ The recovery stress of conventional SMPs is significantly low, to be precise from a tenth of a MPa to several MPa, which is not sufficient as actuators. Studies showed that the recovery stress can be improved by using enthalpy as the primary energy storage mechanism.^33−35^ Clearly, using SMPs with high recovery stress as the matrix is the prerequisite for synthesizing SMP-based syntactic foams with high recovery stress.

Similar to conventional polymers, one of the major problems associated with polymer syntactic foam persists in the poor fire resistance which stimulates the high flammability of the material. It can lead to rapid-fire propagation and heavy loss of property and life, especially in cases where high fire risk is involved, for example, fields like transportation, electronics, and construction industries.^31^ Fortunately, this limitation can be mitigated by utilizing the advantage of SMP with the intrinsic flame-retardant property. Recently, Feng and Li have developed a high-temperature shape memory photopolymer (HTSMP) with intrinsic flame retardancy and excellent thermal stability.^32^ This HTSMP has a high *T*_g_ of 280 °C and displayed a record high recovery stress of 35.3 MPa and energy output of 2.9 MJ/m^3^. Therefore, it can be used as a matrix in preparing flame-retardant syntactic foams.

In recent times, 3D printing technology has attracted a lot of attention as an emerging rapid prototyping technology.^33^ Additive manufacturing (AM), commonly termed 3D printing, is being used in production for many aircraft parts, medical devices, spacecraft components, and consumer products.^34,35^ While polymer 3D printing has been popular, and many soft-polymer inks and printers are commercially available, only limited studies have been conducted to print thermoset shape memory polymers.^36^ To our knowledge, no studies have been conducted to 3D print thermoset shape memory polymer-based syntactic foam.

The objective of this study is to use the HTSMP and glass microballoons to prepare syntactic foams with high mechanical properties, excellent shape memory effect, inherent flame retardancy, and 3D printability. Three different kinds of hollow glass microspheres (HGM) were used. The mechanical, chemical, thermal, and thermomechanical characterizations were conducted. Flame retardancy was investigated. 3D printability of the syntactic foam using a homemade high-viscosity extruder was demonstrated to print complex structures such as a honeycomb, free-standing helical spring, sinusoidal spring, and flower.

## 2 Experimental Section

### 2.1 Raw Materials

The raw materials used in the experiment, tris[2-(acryloyloxy)ethyl] isocyanurate (TAI) with a molecular weight of 423.37 g/mol and photoinitiator diphenyl(2,4,6-trimethylbenzoyl)phosphine oxide (97%) (TPO), were ordered from Sigma-Aldrich. The syntactic foam was fabricated by dispersing glass bubbles in the polymer matrix. Three different kinds of hollow glass microspheres (K1, K15, and K20), with a density of 0.125, 0.15, and 0.20 g/cm^3^, respectively, and an isostatic crush strength of 1.72, 2.07, and 3.45 MPa, respectively, were purchased from Industrial General Store. All the raw materials were used as received without further purifications.

### 2.2 Preparation of the Syntactic Foam

**Scheme 1:**
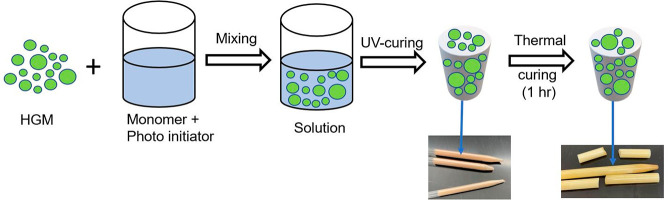
Preparation Process of HGM/HTSMP Syntactic Foam

The syntactic foams were prepared by the following steps (Scheme 1). First, the monomer and photoinitiator, 93 wt % tris[2-(acryloyloxy)ethyl] isocyanurate monomer and 7 wt % photoinitiator diphenyl(2,4,6-trimethylbenzoyl)phosphine oxide, were mixed for 2 h at 100 °C. The 40 vol % glass microspheres were dispersed in this solution by adding a little amount at a time while stirring the solution so that no agglomeration can take place. The microspheres tend to float to the top surface due to the density difference between the glass bubbles and the binder. The solution was then placed in a vacuum chamber at 80 °C for 10 min to remove any air bubbles entrapped into the solution while mixing. After that, the solution was poured into a cylindrical glass mold and moved to the UV chamber (IntelliRay 600, Uvitron International, USA), with wavelength 232 nm and intensity around ∼45 mW/cm^2^ (information provided by the equipment manufacturer), under the irradiation intensity 35% and cured for 10 s. The cylindrical-shaped mold was then turned upside down and cured for another 10 s. This step had been repeated twice to ensure uniform intensity on both sides of the sample during polymerization. To increase the overall conversion, the syntactic foam was then placed in a muffle furnace (FB1315M, Thermo Scientific Thermolyne) at 280 °C for 1 h. The foam was demolded after curing, and the specimens were cut from the bulk for further testing.

### 2.3 Characterization

#### 2.3.1 Surface Visualization

Surface morphologies of the syntactic foams under different conditions such as after curing, after programming, after shape recovery, and the char residue from the flame retardancy test were analyzed by a scanning electron microscope (SEM) (JSM-6610 LV, JEOL, USA). The accelerating voltage used was 15 kV.

#### 2.3.2 Void Volume Determination

The experimental densities (ρ_ex_) of the syntactic foam were calculated as per ASTM D1622-98^37^ by using the ratio of the average mass over the volume of the specimens. Theoretical densities (ρ_th_) were determined by using the standard rule of mixtures approach as follows:
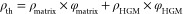
1Here, ρ and φ refer to the density and the volume fraction of polymer (matrix) and hollow glass microspheres, respectively. The ratio of the difference between the experimental and theoretical densities to the theoretical density was used to calculate the void percentage in the sample.

#### 2.3.3 Chemical and Thermomechanical Characterization

A Thermo Nicolet Nexus 670 FTIR spectrometer was used to collect the Fourier transform infrared spectroscopy (FTIR) spectra which collected 512 scans from 650 to 4000 cm^–1^ in the attenuated total reflection mode. Dynamic mechanical performance was performed by using a Q800 dynamic mechanical analyzer (DMA) (TA Instruments, New Castle, DE) in multifrequency strain mode. The approximate sample dimension used was 9 mm × 7 mm × 4 mm. The heating rate was 3 °C/min, the frequency was 1 Hz, and the amplitude was 5 μm. A TGA550 thermal analyzer (TA Instruments, New Castle, DE) was used to perform nonisothermal and isothermal thermogravimetric analysis (TGA) tests from 30 to 800 °C at a heating rate of 10 °C/min in nitrogen environment, and the purging rate of the nitrogen gas was 25 mL/min. The approximate sample mass for TGA testing was 3.5 mg. A Scienta Omicrometer ESCA 2SR X-ray photoelectron spectroscope was used to carry out the X-ray photoelectron spectroscopy (XPS) spectra of the char residue. Raman spectroscopy of the char residue was conducted by a Renishaw inVia reflex with the excitation wavelength 532 nm and a spectral resolution of 1 cm^–1^. The Raman shift was scanned from 500 to 2500 cm^–1^.

#### 2.3.4 Compression Test

The compression properties were investigated by using an Xpert 2610 MTS (ADMET, Norwood, MA) which is equipped with a temperature-regulated oven. At least three cylindrical samples (diameter: ∼5.8 mm; height: ∼6 mm) were used to study the compression behavior of the syntactic foams at room temperature and 275 °C. For all the compression tests, the compression rate was 0.5 mm/min.

#### 2.3.5 Shape Memory Effect Test

The shape memory effect tests were performed following the procedure reported by our group.^38−40^ In the test, the cylindrical sample with an initial height *h*_0_ was placed in between the MTS clamps and heated to 275 °C for 30 min. Once the thermal equilibrium was achieved, the sample was compressed to a certain height (*h*_1_) at a compression rate of 0.5 mm/min, and this strain was maintained for 10 min; the sample was then rapidly cooled to room temperature by spraying water with a wash bottle. After removal of the load, the height of the programmed sample was measured and recorded as *h*_2_. The programmed sample was put in an oven and heated for 60 min at 275 °C. The height of the recovered sample was measured as *h*_3_. The whole process is shown in Figure S1. The shape fixity ratio (*F*) and the recovery ratio (*R*) can be calculated following eqs 2 and 3, respectively:
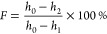
2
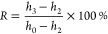
3

#### 2.3.6 Stress Recovery Test

The recovery stress of the syntactic foams was obtained according to our group’s previous report.^41^ In brief, the sample was programmed according to the procedure described in the previous section. To avoid the thermal expansion of the metal fixtures, the MTS fixtures were heated in the attached oven at 275 °C for 60 min. After that, the programmed sample was rapidly inserted between the fixtures in such a way that the sample became confined in full (under zero recovery strain). The recovery stress was recorded as a function of time.

#### 2.3.7 Flame Retardancy Test

The flame retardancy property of the syntactic foams was evaluated by conducting a simple burning experiment. For this experiment, a square plate sample of dimension 100 × 100 × 5 mm^3^ was prepared and placed horizontally. The sample was ignited by a flame torch until the specimen burned completely. The whole combustion process was recorded by a camera, and a thermocouple was used to record the temperature change on the backside. The char residues of the foam samples were collected for further analysis.

#### 2.3.8 Rheological Studies

The rheological studies were performed by using a Discovery HR 30 rotational rheometer manufactured by TA Instruments (New Castle, DE). The geometries of the test fixtures were parallel disks with a diameter of 25 mm for all measurements. A frequency sweep test was conducted at room temperature, and the scanning frequency range was from 0.1 to 100 rad/s. The temperature sweep of the foams was also performed at a heating rate of 3 °C/min from 25 to 100 °C, and the angular frequency was kept constant (10 rad/s). The conditioning time and the sampling time for data acquisition were both set to 3 s.

## 3 Results and Discussion

### 3.1 Void Volume of Syntactic Foam

**Figure 1:**
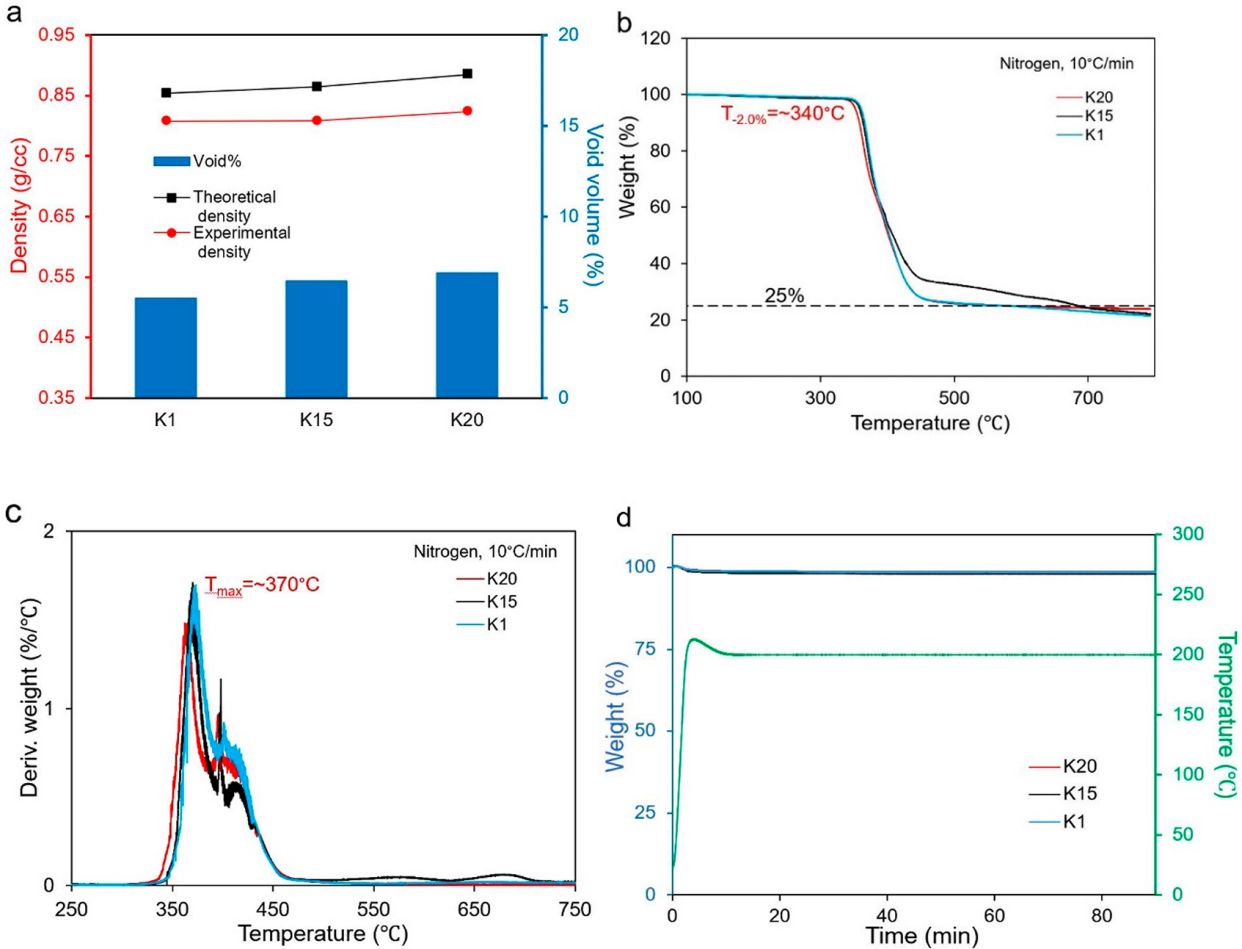
(a) Theoretical and experimental densities along with associated void volume percentage of the syntactic foams. (b) TG curves of the syntactic foams under inert nitrogen weight % vs temperature. (c) Derivative weight vs temperature. (d) TG curve of the syntactic foam isothermal at 200 °C for 2 h.

In Figure 1a, the experimental densities (ρ_ex_), theoretical densities (ρ_th_), and the void volume percentage in the syntactic foams are presented. The experimental densities are lower than the theoretical densities of all three syntactic foams. The reason behind the observed discrepancy is due to the entrapment of air bubbles in the mixture. From the literature review, it is obvious that the mean HGM sizes follow the order K1 > K15 > K20, which means the syntactic foam containing K20 HGM has more smaller particles within the foam.^42−44^ In general, the presence of smaller particles introduces more void spaces within the composite. K20 HGM filled syntactic foam seems to have a higher void volume percentage (6.9%) than that of K1 (5.5%) and K15 (6.5%) filled foams. This relates to the increasing trend of void volume percentage with the decreasing microsphere sizes. Previous studies also show that the syntactic foam prepared by using the conventional method, including the manual mixing of the binder and filler, followed by degassing and curing, can have porosity ranging from 3.0 ± 0.69% to 6.5 ± 0.56%.^45−47^ This type of void space or entrapped air is unavoidable in systems containing small particles and the presence of this kind of void space tends to aggravate the mechanical properties of the polymer composite and syntactic foams.^48^ The density of composite foam may be higher than that of traditional open-cell foams. However, with the same type HGM and the same HGM volume fraction (40%), the density is very similar among our foams and the foams by other epoxy matrixes. Table S1 shows the density comparison between different syntactic foams.

### 3.2 Thermal and Chemical Characterization

Thermogravimetric analysis (TGA) of the syntactic foams was performed under a nitrogen atmosphere in nonisothermal mode to study the thermal stability of the syntactic foams. Figure 1b shows that the degradation profile of the three syntactic foams remains the same except for the significant increase in the char content of the foam containing K15 HGM. The initial decomposition temperature corresponding to 2% weight loss of the syntactic foams was found to be around 340 °C, and the temperature at which the maximum rate of weight loss occurs is around 370 °C (Figure 1c), which indicates higher thermal stability than many conventional thermoset polymer-based syntactic foams. For example, Hu et al. reported a UV-heat-cured epoxy resin E-44 (6101)-based syntactic foam. The temperature corresponding to 5% weight loss of the syntactic foam is around 290 °C.^49^ The initial decomposition temperature corresponding to 5% weight loss of the syntactic foam containing 40 vol % HGM and DER 332 epoxy resin was found to be around 340 °C.^50^ Comparatively, the maximum rate of weight loss of the syntactic foam containing K1 HGM is slightly higher than that of the other two syntactic foams. Figure 1b also characterizes the charring capability of the syntactic foams containing different HGMs. In terms of charring ability, the performances of the syntactic foam containing K1 and K20 HGMs follow a similar pattern, and the foam containing K15 HGM is found to be better than the other two. At 500 °C the char residue of the syntactic foam containing K1 and K20 is 25 wt %, whereas the char residue of the foam containing K15 HGM is around 30 wt %. The residual percentage value and the corresponding temperature value indicated good charring ability during thermal decomposition. The char residue percentage of the syntactic foam is higher than the pure polymer (20 wt %), which is indicative of the improved fire retardancy of the foam. The reason behind this is the presence of the HGM (40 vol %) in the syntactic foam which is incombustible, and the combustible part of the foam is the polymer matrix. Feng and Li have performed a detailed analysis on the thermal decomposition of the pure polymer.^32^ The flame-retardant property of the syntactic foam will be discussed in further detail in a later section. Furthermore, the thermal oxidative stability of the syntactic foams under the nitrogen atmosphere in isothermal mode had been studied where the sample was kept isothermally at 200 °C for 2 h, and the isothermal TG curve is shown in Figure 1d. After heating for 2 h at 200 °C, the associated weight loss was ∼1.6% for all the syntactic foams, which may include a portion of absorbed water. The results of the nonisothermal TG curves are reported in the Supporting Information (Table S2). From the thermal stability analysis, clearly, all the syntactic foams, irrespective of the type of microspheres, have excellent thermal stability which can be beneficial in services where high temperature and long-term stability are required.

FTIR spectra were acquired to identify the evolution of the molecular structures of the syntactic foams containing different HGMs. Figure S1 shows the transmittance spectra of the syntactic foams and the monomer + initiator solution, in which peaks are identified at 1600–1800, 900–1500, and 700–800 cm^–1^. A small deviation is observed around 2950 cm^–1^ which may be attributed to CH_2_ stretching. The peak here is broadened and shifted a little bit toward the lower wavenumber which may also indicate hydrogen-bonding interaction between the filler HGMs and the polymer matrix^51−53^ The peaks in the range 1600–1800 cm^–1^ are related to the carbonyl stretching, and the shoulder around 1720 cm^–1^ may have resulted from carbonyl stretching in the amorphous domain.^28^ The peak at 1450 cm^–1^ corresponds to the C=C stretching and CH_3_ deformation vibration mode. Another peak has been observed within the range 700–800 cm^–1^ which may be related to the C=C groups. All three syntactic foams show similar kinds of transmittance spectra. When compared to the FTIR spectra of the monomer and initiator mixture solution, it seems that the peak intensities are much decreased, and this shifting of peaks suggests polymerization and interaction between the polymer matrix and the filler HGMs.

### 3.3 Thermomechanical and Mechanical Characterization

**Figure 2:**
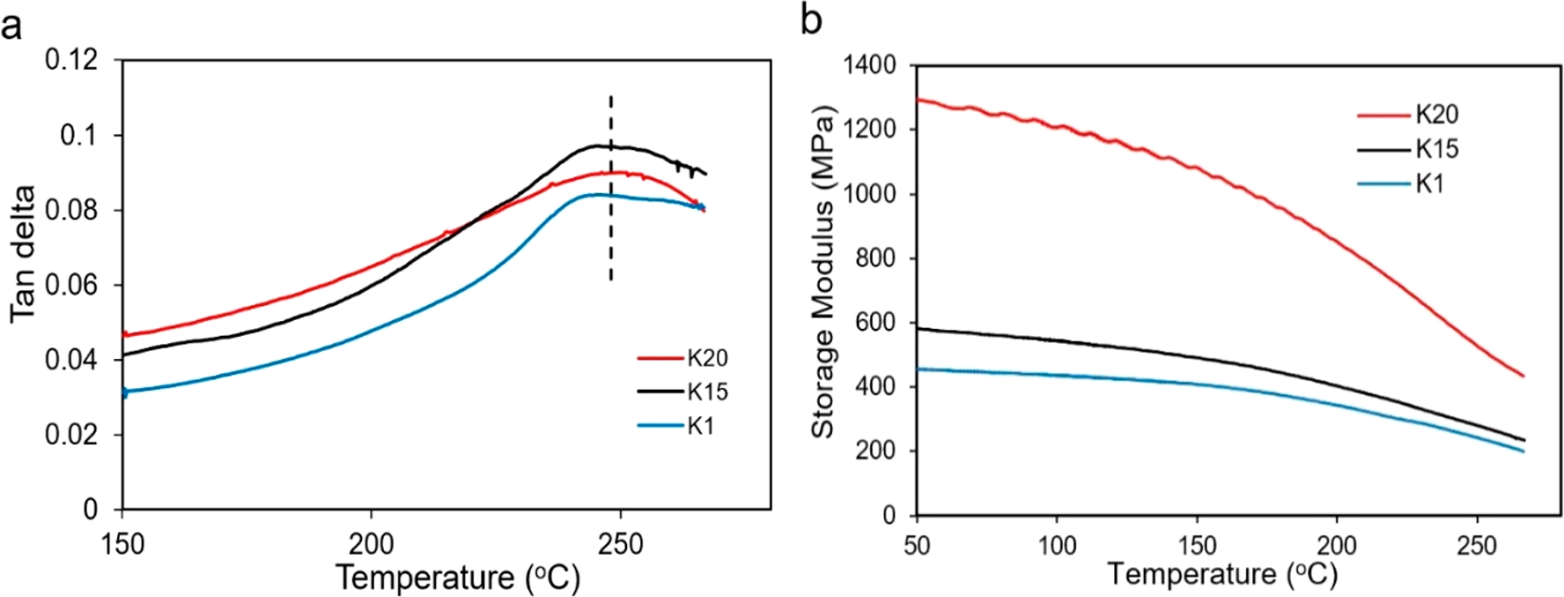
(a) Tan delta and (b) storage modulus curves of the syntactic foams containing different HGMs.

For shape memory thermoset polymer, the glass transition temperature (*T*_g_) is an important indicator. Similarly, the *T*_g_ value controls the shape recovery process of the thermoset shape memory polymer-based syntactic foam. Dynamic mechanical analysis (DMA) was performed to characterize the *T*_g_ values of the syntactic foams, as shown in Figure 2a,b. From our previous study, the *T*_g_ value of the pure polymer (UV + 3 h thermally cured) is 280 °C, and when the postcuring time was decreased from 3 to 1 h, it leads to a decrease in the *T*_g_ value from 280 to 269 °C. In this study, the syntactic foams were thermally cured for 1 h after the short-time UV exposure. The tan delta peak in the tan delta vs temperature plot generally shows the glass transition region. The *T*_g_ values of the syntactic foam samples containing K1, K15, and K20 HGMs are 245, 248, and 250 °C, respectively. Compared to the pure polymer (UV + 1 h postcure), the *T*_g_ value is decreased for the syntactic foams. In general, types of chain segments, cross-linking density, and interaction between the thermoset segments influence the *T*_g_ value. In this case, the decrease in the tan delta peak to a lower temperature is also caused by incorporating HGM in the polymer matrix. This *T*_g_ value is higher compared to that of other syntactic foams^16,17,28^ and can be beneficial for high trigger temperature required fields. The obtained syntactic foams show a broad glass transition region instead of a sharp peak in the tan delta curve which is typical for photopolymers. The storage modulus vs temperature curves of the syntactic foams are presented in Figure 2b. The storage modulus represents the elastic response of the polymer. At room temperature, the storage modulus of the syntactic foams containing K1, K15, and K20 HGMs are 458, 588, and 1300 MPa, respectively. Even at the temperature where the upper limit of the tan delta peak exists (∼250 °C), the storage modulus is found to be 241, 278, and 525 MPa, respectively. The result suggests that K20 syntactic foam is stiffer among the three foams.

**Figure 3:**
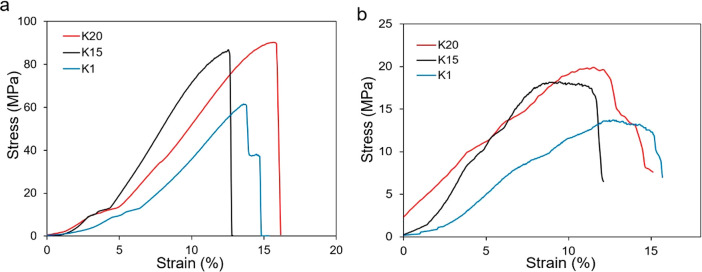
Mechanical testing of the syntactic foams: (a) room temperature and (b) high temperature.

**Figure 4:**
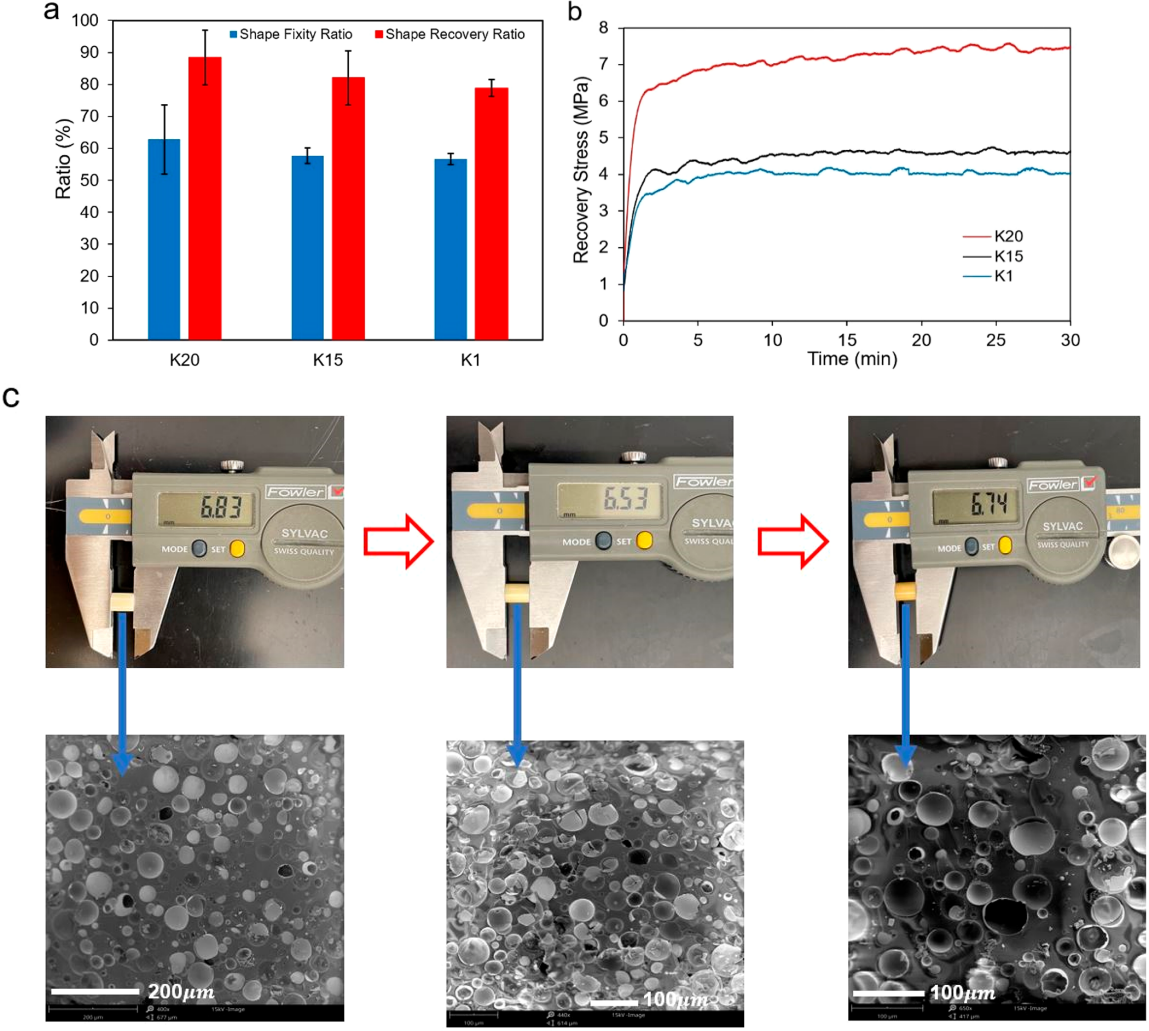
(a) Shape memory parameters of the syntactic foams. (b) Fully constrained stress recovery profile of the syntactic foams. (c) Photos (top) and corresponding SEM images (bottom) of the syntactic foam sample containing K20 HGM showing the original–compression programming–recovery cycle.

The compressive strength of the syntactic foams was studied which provided the fundamental information about the mechanical performance. Typically, the thermoset polymer-based syntactic foams exhibit a yielding–densification–strain hardening–fracture behavior at glassy state. In the case of syntactic foam filled with HGM, at a certain strain, the glass microspheres fracture, which results in opening the enclosed porosity for the compressing material to occupy. Hence, the stress-plateau region with strain appears. After a certain point, once a large number of microspheres are crushed and compacted, stress begins to increase steeply which eventually results in failure at a certain strain. Figure 3a shows the compressive stress–strain curves of the syntactic foams at room temperature, which increased linearly with strain but do not stick to the typical yielding–fracture behavior. When the strain reached around (13–14%), failure occurred, exhibiting the internal brittle rupture behavior. The reason behind this is due to the comparatively brittle behavior of the polymer matrix. As shown in Figure 2d in ref (32) the pure polymer fractured at about 36% compressive strain at room temperature without the typical postyielding strain softening and plastic flow for typical glassy polymers. Another reason is that at room temperature the polymer is stiffer than the HGMs. Therefore, the syntactic foam can be treated as a soft particle dispersed in a hard matrix. Under uniaxial compression, hoop tensile stress concentration, which is perpendicular to the compression direction, occurs at the polymer/HGM interface, leading to tensile fracture of the polymer matrix. As a result, the matrix fractures before crushing and densification of the HGMs, which can be further validated by the SEM images in Figure 4c. Not many HGM fractures can be observed. As a result, the typical yield–densification–strain hardening–fracture for conventional polymeric syntactic foam did not show here. This kind of quasi-linear stress–strain relationship has also been observed in the studies reported in the literature.^54,55^ In this study, the syntactic foam containing K20 HGM exhibited the highest compressive strength (81.8 ± 7.5 MPa), followed by K15 HGM (77.8 ± 7.0 MPa) and K1 HGM (59.8 ± 8.0 MPa). Compared to most of the reported syntactic foams in the literature, the compressive strength of the foam is relatively higher.

To provide reference strain for subsequent programming study, the compression tests of the syntactic foams were also performed at rubbery state (*T*_g_+ 25 °C), as shown in Figure 3b. For obvious reasons, the compressive behavior of the foam is slightly different from that at the room temperature. Both do not display any densification zone. The high-temperature compressive stress–strain curve has no yield point, and the postpeak stress decreased gradually instead of sharp fracture seen at room temperature. The peak compressive stress is 14.6, 17.7, and 18.6 MPa for the foams containing K1, K15, and K20 HGM, respectively, and fractures at a failure strain around 10%.

### 3.4 Shape Memory Effect

To evaluate the shape memory properties, the compressive deformation at rubbery state was selected as a base parameter. Figure S2 illustrates a compression programming–recovery cycle. In brief, the first step is the programming in which the sample was compressed to a certain strain (in this case, 8% for all the foams) at 275 °C, and this stress is maintained for at least 10 min to achieve stress–relaxation. After that, the sample was cooled rapidly to room temperature by using a wash bottle while keeping the compressive strain constant. After the load removal and springback, the shape fixity ratio of the foams containing K1, K15, and K20 HGM are found to be 56.7, 57.6, and 62.8%, respectively, whereas the shape fixity ratio of the pure polymer (UV + 3 h postcured) is 58% (Table S3). The programmed foam was then heated to 275 °C in a heating chamber and recovered to the height close to its original. Though the pure polymer shape recovery ratio was 93.1%, the recovery ratios of the syntactic foams containing K1, K15, and K20 HGMs were found to be 78.85, 82.06, and 88.46%, respectively. This indicates that the recovery ratio of the syntactic foam is lower than the pure polymer, and K20 HGM has the best shape memory property among the foams. The shape fixity ratio and the recovery ratio of the foams are shown in Figure 4a. Figure 4c shows the digital images of the compression–programming–recovery cycle of the syntactic foam containing K20 HGM along with the corresponding scanning electron microscopy (SEM) images of the fractured surfaces. From the SEM observations, it is evident that the syntactic foam exhibits a composite structure with HGM (40 vol %) dispersed in the polymer matrix. The polymer matrix cracked with few fractured microspheres. Void spaces have been observed in the SEM images. This validates the fact that the interfacial bonding between the HGM–polymer matrix system is comparatively weak, and interfacial debonding may occur under external force, resulting in decreased strength. The different strengths of the HGMs and the introduction of the voids during mixing are also contributing factors here. No significant difference had been observed among the morphologies of the three different syntactic foams except for the size variation. Similar images had been obtained for the other two syntactic foams (Figure S3); the K20 syntactic foam is used as a typical example.

The stress recovery performance was studied according to the scheme illustrated in Figure S4, which explains the procedure to produce recovery stress during constrained shape recovery test. During the free shape recovery test by heating, the recovery stress remains zero, that is, free from any external constraint. In the case of the fully constrained shape recovery test, the recovery strain is set at zero; that is, shape recovery is not allowed. At a temperature higher than the glass transition temperature, the programmed sample tends to recover to its original shape even under external constraint. Because of the external constraint, the shape recovery is not allowed. As a result, the sample is equivalent to the case under a pushback force. This pushing force is known as the recovery force.^56^ To evaluate the recovery stress performance, the programmed sample was fully constrained at rubbery state temperature (*T*_g_ + 25 °C) with zero recovery strain allowed. From Figure 4b, the maximum recovery stress for K20 foam, K15 foam, and K1 foam is 6.8, 4.7, and 3.8 MPa, respectively, and this stress had remained stable for 30 min during the experiment. It is noted that this recovery stress level of the syntactic foam is higher than most thermoset shape memory polymers without the microspheres.^57,58^ Among the three syntactic foams, K20 foam has the highest recovery stress due to the higher rubbery modulus of this foam.

### 3.5 Flame Retardancy Property

**Figure 5:**
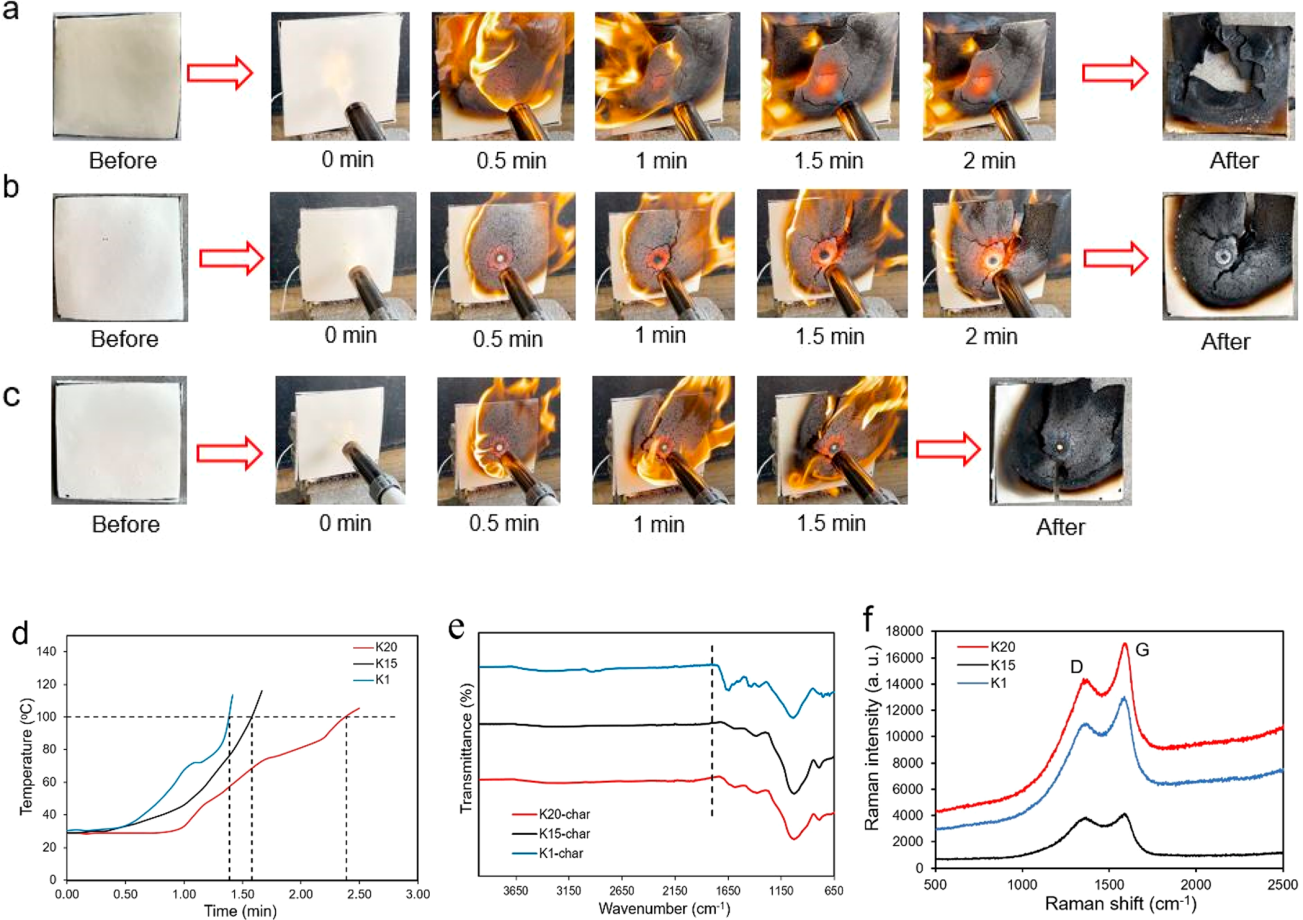
Flame retardancy: (a), (b), and (c) combustion performance of syntactic foams containing HGM K20, K15, and K1, respectively. (d) Temperature–time curve on the backside of the syntactic foam plates. (e) FTIR spectra. (f) Raman spectra of the char residues.

From the thermogravimetric analysis in section 3.2, it is obvious that the syntactic foams have excellent thermal stability. The polymer matrix used in the foam has an acceptable flame retardancy due to the incorporation of 7 wt % TPO (photoinitiator).^32^ Incorporation of the incombustible hollow glass microspheres (40 vol %) can further enhance the flame-retardant property of the foam. The flame-retardant property of the syntactic foam can be useful as a layer of protection in the mass and heat transfer process which is a key requirement for lightweight structures. Figures 5a, 5b, and 5c display the combustion process of the syntactic foam plates containing microspheres K20, K15, and K1, respectively. In the beginning, the plate sample was ignited, and within a few seconds the plate started to burn vigorously. A black char layer was formed on the fire area which propagated with time. The flame torch was not removed the whole time. After 1 min of burning, crack was initiated in the sample containing K1 and K15 microspheres. The sample containing K20 microspheres seemed to burn a little longer (1.5 min) before crack had been identified. After burning for around 3 min, a compact char layer was formed on the surface, which implies the flame-retardant property of the foam. The fire stopped as soon as the flame torch was removed. The temperature reading from the thermocouple on the back surface illustrates the temperature change of the foam plate with time (Figure 5d). As shown in the figure, the temperature of the plate containing K1 and K15 HGM increased rapidly within 1.5 min, whereas the foam plate containing K20 microspheres took 2.5 min to reach 100 °C, suggesting the comparatively better performance of the foam containing K20 HGM. It is noted that the whole combustion experiment was done outside where wind gust was also affecting the pattern of the burning flame. Hence, the deviation in the curve of the sample containing K20 and K1 has been observed. The char residues from the combustion experiment were collected for further assessment. Figures 5e and 5f display the Fourier transform infrared (FTIR) spectra and the Raman spectra of the char residue, respectively. In Figure 5e, peaks are observed mostly at 650–1800 cm^–1^. The peak position and intensity varied depending on the type of HGM in the syntactic foam. Compared to the other two foams, a peak around 1650 cm^–1^ has been observed in the profile of the K1 HGM syntactic foam, which may be assigned to the C=O stretching. Raman spectroscopy (RS) is used to characterize the graphitization degree of carbonaceous substances after combustion. In Figure 5f, intensities of the D and G bands relate to amorphous carbon and graphitized carbon, respectively. Typically, the Raman spectra of the carbon signals show the D and G bands at 1360 and 1580 cm^–1^, respectively. The graphitization extent of the residue and the stability of the char structure are characterized by the ratio of the intensities of the D and G band (*I*_D_/*I*_G_). A lower value of the intensity ratio indicates a more stable char structure with more intense graphitization and better flame retardancy.^59^ The ratio *I*_D_/*I*_G_ of the syntactic foams containing K1, K15, and K20 HGM is 0.84, 0.94, and 0.83, respectively, which implies the higher graphitization degree of the residue in the syntactic foam containing K20 HGM.

**Figure 6:**
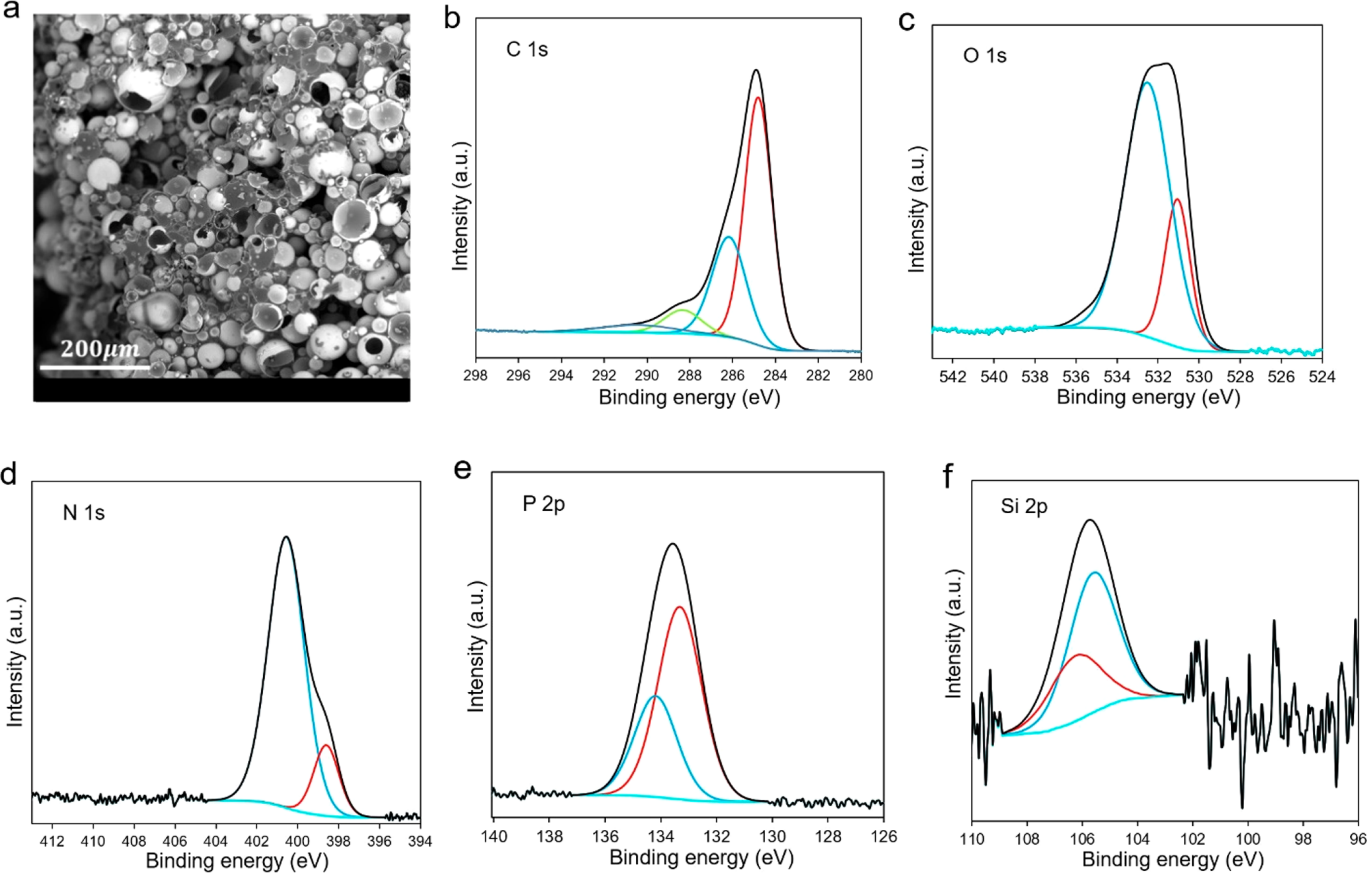
(a) SEM observations of the char residue of the foam containing K20 HGM. High-resolution (b) C 1s, (c) O 1s, (d) N 1s, (e) P 2p, and (f) Si 2p XPS spectra of the char residue of the syntactic foam containing K20 HGM.

To explore the flame-retardant mechanism and char structure, SEM and XPS characterizations for the char residues of the syntactic foams were performed, and the results are summarized in Figure 6. Figure 6a shows the SEM observations of the char residue of the foam containing K20 HGM. From the visual appearance, the char residue is intact and continuous, but the SEM image shows that a major part of the foam burned during the combustion process is the polymer matrix. The microspheres present in the char are mostly intact and smooth along with some fractured HGMs. This validates the fact that the incombustible nature of the microsphere led to this type of combustion and enhanced the flame retardancy of the foam compared to the pure polymer. Similar kinds of SEM images have been obtained for the other two foams (Figure S5). XPS spectra of the char residue containing K20 HGM are reported in Figure 6b–f, and the other two foam analyses can be found in Figures S6 and S7. The char residue of the foam mainly contains C, N, O, P, and Si, and the high-resolution X-ray photoelectron spectroscopy (XPS) spectra of these elements demonstrate the surface chemistry and the binding characteristics. The C_1s_ spectrum peaks are centered at 284.5, 286.6, and 288.7 eV, which could be assigned to C–C and C–H of the aliphatic and aromatic species, C–O group, and C=O group, respectively.^52^ In Figure S6 (K1 HGM syntactic foam char), one C_1s_ peak has been observed at 282.5 eV, which may be attributed to the Si–C group.^52,60^ The O_1s_ spectrum shows two peaks at 531.1 and 532.5 eV, in which the first one may be related to the P=O or C=O groups and the second one may be assigned to C–O–C groups, respectively.^52^ In the case of K1 foam char, only one peak at 531.7 eV has been noticed. The N_1s_ spectrum displays that the bind energy attributed to the stable C–N bond in the six-membered ring in isocyanurate species and the N–P bond in the char residue to be 400.5 and 398.6 eV, respectively.^61^ The P_2p_ spectrum has two peaks at around 133.3 eV (P=O) and 134.2 eV (P=N).^52,62^ In addition, the peaks in Si_2p_ spectra at 105.6 and 106.2 eV may be attributed to the silicone oxides structures.^63^ On the basis of these findings and the SEM images, it is evident that when the syntactic foams are ignited, the thermally stable isocyanurate and phosphine oxide structures mostly contribute to the formation of the char of the syntactic foam. This can delay the thermal decomposition and hinder the heat transfer from the combustion area to the substrate. Also, the barrier-like char layers slow down the spread of combustible pyrolysis volatiles and reduce the fire hazard. This section summarizes the qualitative study of the flame-retardant property of the syntactic foams. However, some classic flame-retardant tests, such as limiting oxygen index, UL-94, cone calorimetry experiments, and so on,^64−66^ can be performed to further quantify the flame-retardant properties in our future studies.

### 3.6 3D Printing

**Figure 7:**
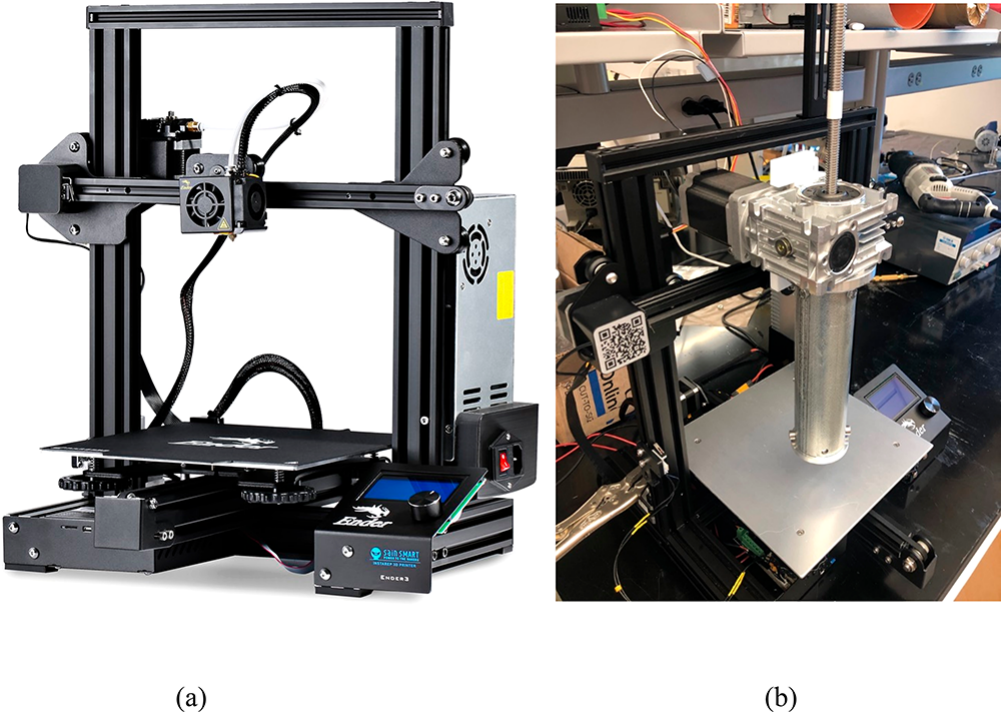
(a) Creality Ender 3 filament printer. (b) The modified homemade 3D printer.

In recent years, three-dimensional (3D) printing technology has attracted a lot of attention. In this study, an extruder (like a direct ink writing (DIW)) type printer was assembled in our lab to evaluate the printability of the developed syntactic foams. The high-viscosity 3D printer was built from a modified Creality Ender 3 filament printer. The original filament print head and heating print-bed apparatuses were removed and replaced with a custom high-viscosity extruder and flexible film heater, respectively. The custom extruder used a power screw which was turned by a larger NEMA 23 stepper motor and pushed an aluminum plunger down the extruder’s body tube. It was powered by using a benchtop power supply and was controlled via an Arduino Uno and a microstep controller. Different aluminum nozzles can be screwed onto the body tube’s end depending on desired diameter. If the printing material needs to be heated, the film heater can be wrapped around the body tube and controlled via the Ender 3’s integrated print bed temperature controls. A custom UV LED ring, which was powered by the printer’s main power supply, was taped to the nozzle’s end while printing photocurable materials. Print parameters were controlled via Slic3r software due to its open-ended design. In this study, the print rate was around 5 mm/s. The layer height was set to be the same as the nozzle’s diameter, here 3 mm. The printing was conducted at room temperature while the foam inside the tube was preheated to about 60 °C before loaded into the tube. The printing parameters were then transferred to the printer via a memory card and run like a filament print. The original 3D printer is shown in Figure 7a, and the assembly of the modified 3D printer is shown in Figure 7b.

**Figure 8:**
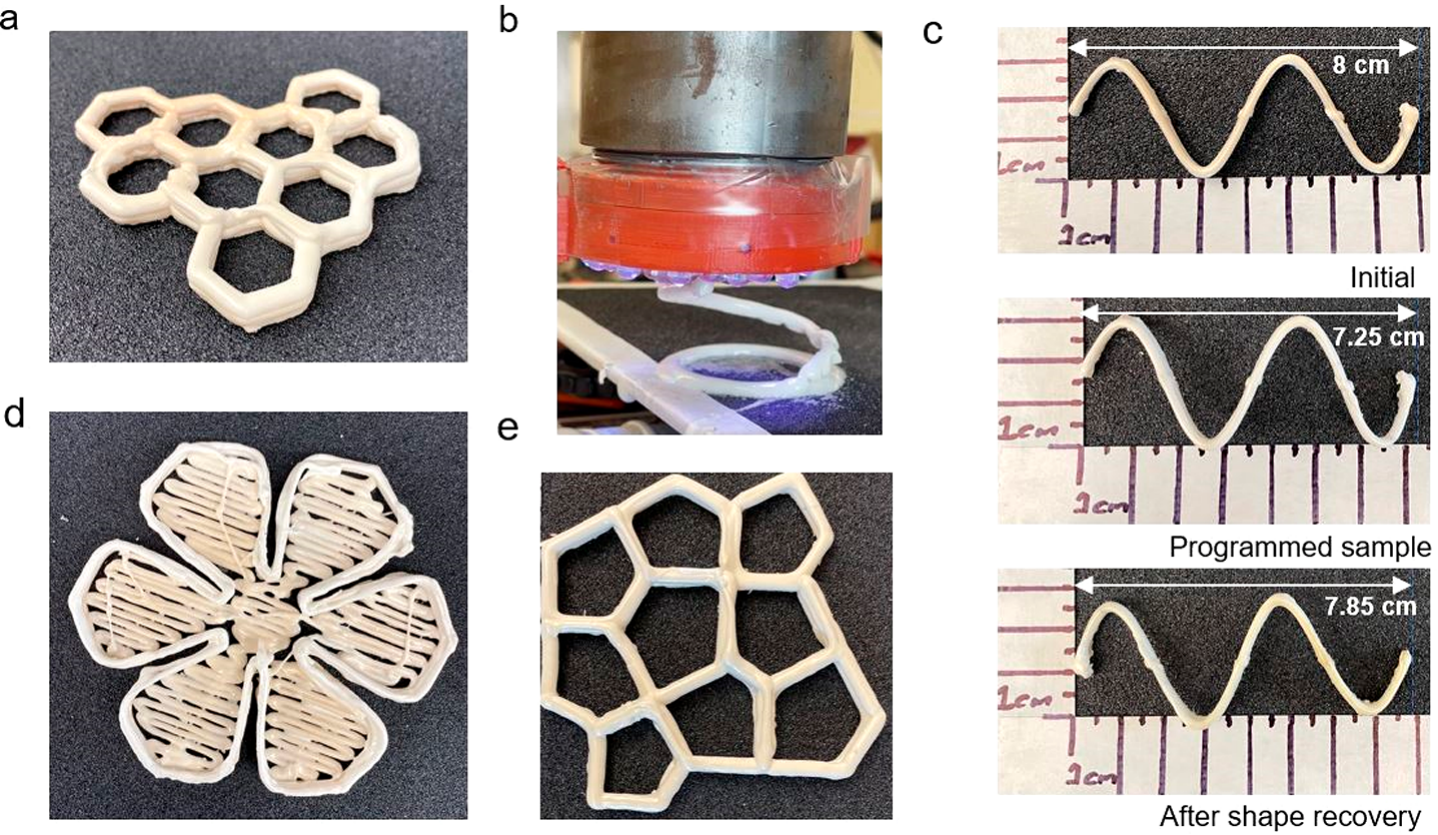
(a) 3D printed honeycomb shaped syntactic foam containing K20 HGM. (b) Printing of a free-stranding helical spring by the 3D printing extruder. (c) Shape memory effect test of sinusoidal sample. (d) 3D printed flower. (e) 3D printed lattice shaped syntactic foam containing K20 HGM.

**Figure 9:**
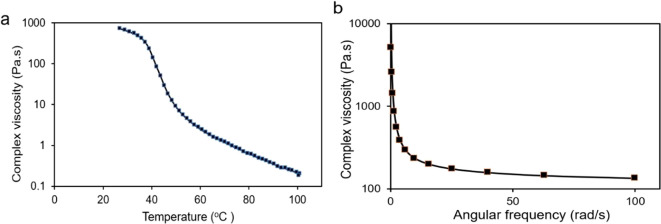
Change of complex viscosity with (a) temperature and (b) angular frequency.

With this homemade 3D printer, the 3D printability of the syntactic foam with K20 microspheres has been evaluated, and several shapes such as honeycomb, sinusoidal curve, cylindrical, and so on have been printed (Figure 8). The reason we used this homemade extruder (like a direct ink writing (DIW) printer) instead of the digital light processing (DLP) printer in our lab^36^ is that the syntactic foam has very high viscosity. Figure 9a shows the complex viscosity of the K20 foam with temperature. It is seen that this foam has very high viscosity at room temperature. While adding diluent can reduce the viscosity and making it compatible with the DLP printer, the diluent may compromise the mechanical and functional properties of the foam. However, as shown in Figure 9b, with the increase in angular frequency, the complex viscosity of the foam reduces, leading to shear thinning behavior. The shearing thinning behavior makes K20 foam 3D printable. Figure 8c shows the preliminary study of the shape memory effect of a 3D printed two-layer sinusoidal structure. The sinusoidal syntactic foam of length (*h*_0_) was heated at 275 °C for 5 min, followed by compression manually by using a press. The sinusoidal foam was then left to cool, and the length (*h*_2_) was recorded after cooling and load removal. The foam sample was then heated in an oven at 275 °C for 5 min for free recovery. The length of the recovered foam sample (*h*_3_) was recorded, and the shape recovery ratio is found to be 80%. For cylindrical samples containing K20 HGM, the recovery ratio is 88.46%. Though this was a very crude estimation, still it shows similar shape memory effect. Our study proves that this syntactic foam containing the K20 HGM is 3D printable, and it shows the shape memory effect. In general, insufficient recovery stress is a disadvantage for developing and 3D printing high-performance lightweight shape memory polymers. Most thermoset SMP exhibit recovery stress less than 2 MPa or even 1 MPa in rubbery state, and as a result the energy output during shape recovery becomes limited.^58^ The syntactic foam studied here possesses high recovery stress (6.8 MPa for syntactic foam with K20 HGM) at the rubbery state, much higher than the shape memory polystyrene-based syntactic foam (0.22 MPa),^18^ and shape memory polycaprolactone-based syntactic foam (0.19 MPa).^31^ 3D printability along with the shape memory effect and record-high recovery stress can be beneficial in different types of applications in different sectors such as soft robots, deployable structures, electronics, and self-healing. For example, based on the close-then-heal (CTH) strategy for healing wide-opened cracks,^6,18,25^ high recovery stress is a necessary condition to bring the fracture surfaces in touch by the recovery stress. This type of foam can be also utilized to print composite sandwich structures, and the lightweight property can be beneficial for many weight-sensitive applications.

## 4 Conclusions

In conclusion, we reported the preparation and mechanical and shape memory property study of a thermoset shape memory polymer-based syntactic foam, which exhibits flame-retardancy property, record high recovery stress of 6.8 MPa, and shear thinning and 3D printability. We studied three different syntactic foams based on an HTSMP matrix and three hollow glass microspheres (K20, K15, and K1). From the results presented in this paper, the following conclusions are summarized:

- All three syntactic foams have high glass transition temperature and thermal stability.
- The compression tests showed excellent compressive properties of the syntactic foams. Also, the compressive strength varies with the size and type of the HGM, indicating the dependency of the foam properties on HGM stiffness and size. Compared with conventional polymer-based syntactic foams, which can be compressed up to more than 50% strain under uniaxial compression, this HTSMP based foam possesses lower ductility, resulting in a stiff structure.
- The foams show very good shape memory effect. In particular, their recovery stresses are the record for SMP-based syntactic foams reported in the literature.
- The syntactic foams studied here show excellent flame-retardant performance. Although it may not extinguish the fire completely, it can buy valuable time for firefighters to rescue lives and properties before structural collapse.
- We demonstrated the 3D printability of the foam by an extrusion type of printer with several different structures, including a free-standing structure.
- The excellent fire retardancy and 3D printability, together with the high mechanical strength, good shape memory effect, record-high recovery stress, and intrinsic lightweight, make these foams a potential candidate for lightweight structures and devices in several industrial sectors.

## Supporting Information Available

Characterization information including FTIR, SEM, XPS, TGA, density comparison, shape memory characteristic parameters, schematic diagram of shape memory effect, and stress recovery test (PDF)
Video of the combustion process of the syntactic foam containing K15 HGM (MP4)
Video of the 3D printing of honeycomb shape (MP4)
Video of the 3D printing of flower shape (MP4)
