# Supplementary material for: A Thermoset Shape Memory Polymer-Based Syntactic Foam with Flame Retardancy and 3D Printability
Source: ACS Appl Polym Mater. 2022 Jan 26;4(2):1183–95. doi: 10.1021/acsapm.1c01596 (PMC8845046; doi:10.1021/acsapm.1c01596)
Supplement: Supplementary file 1 — ap1c01596_si_001.pdf [file ap1c01596_si_001.pdf]

Supporting Information

A thermoset shape memory polymer based syntactic foam with flame retardancy and 3D printability

Rubaiyet Abedin[a], Xiaming Feng[b], John Pojman, Jr.[b], Samuel Ibekwe[a], Patrick Mensah[a], Isiah Warner[c], Guoqiang Li[a,b,*]

[a]*Department of Mechanical Engineering, Southern University and A & M College, Baton Rouge, LA 70813, USA*
[b]*Department of Mechanical & Industrial Engineering, Louisiana State University, Baton Rouge, LA 70803*
[c]*Department of Chemistry, Louisiana State University, Baton Rouge, LA 70803, USA*

**Corresponding author. E-mail: lguoqi1@lsu.edu; Tel.: 001-225-578-5302*

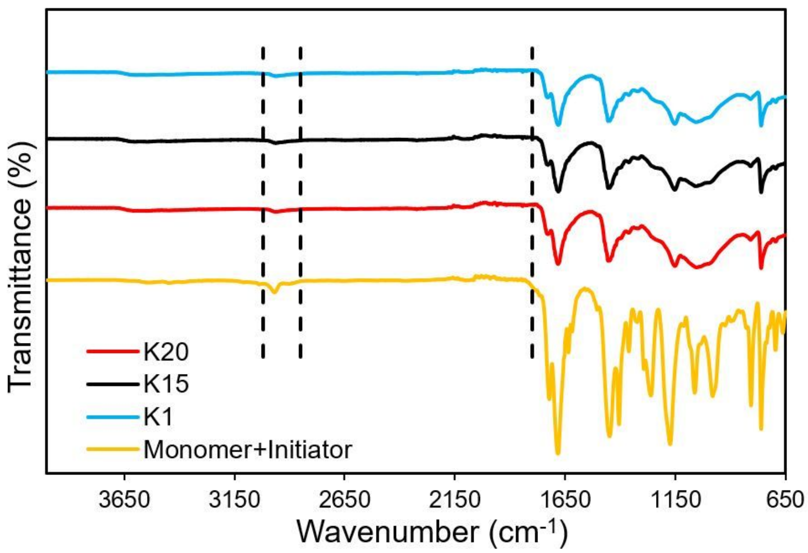

Figure S1. FTIR spectra of the syntactic foams containing different HGMs (K1, K15, K20) and monomer + initiator solution

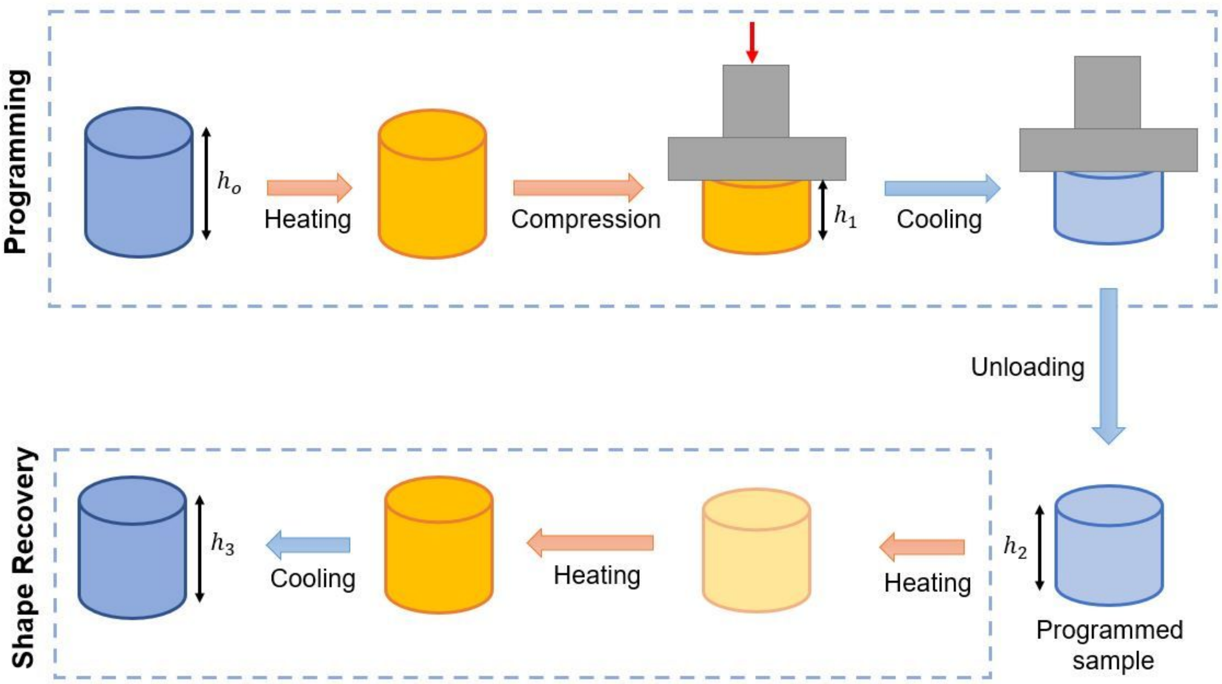

Figure S2. A schematic diagram of compression programming-recovery cycle of syntactic foam sample.

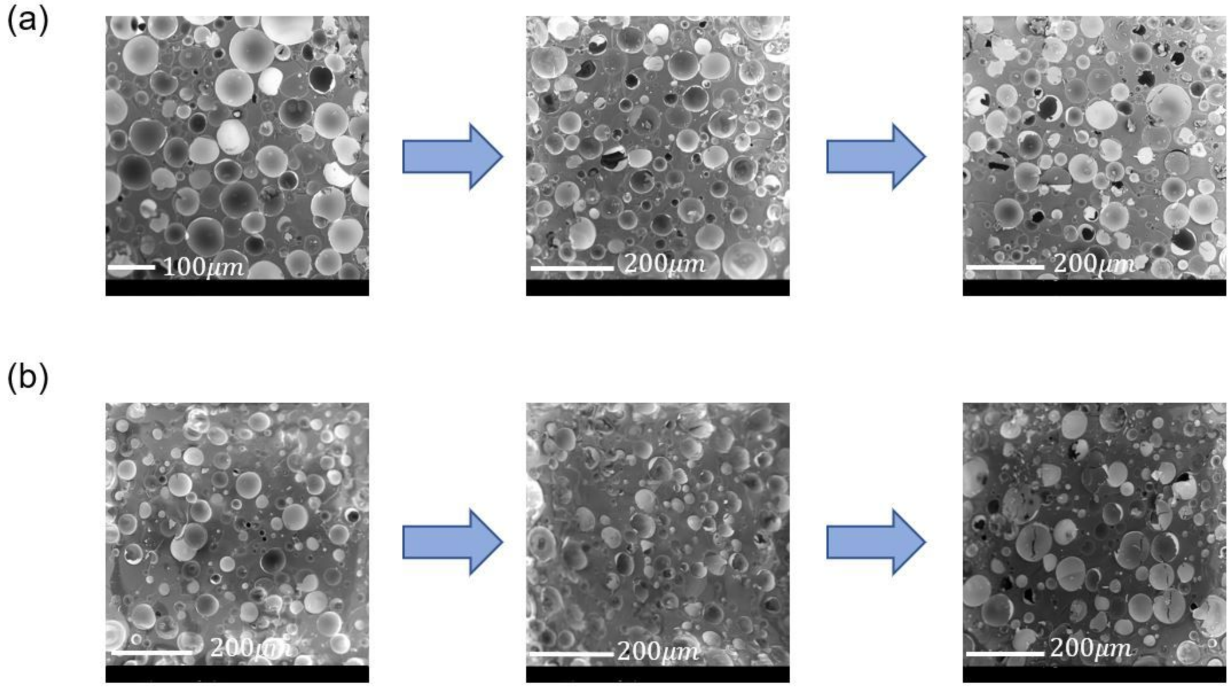

Figure S3. SEM images of the syntactic foam sample containing (a) K1 (b) K15 HGM showing the original-compression programming-recovery cycle

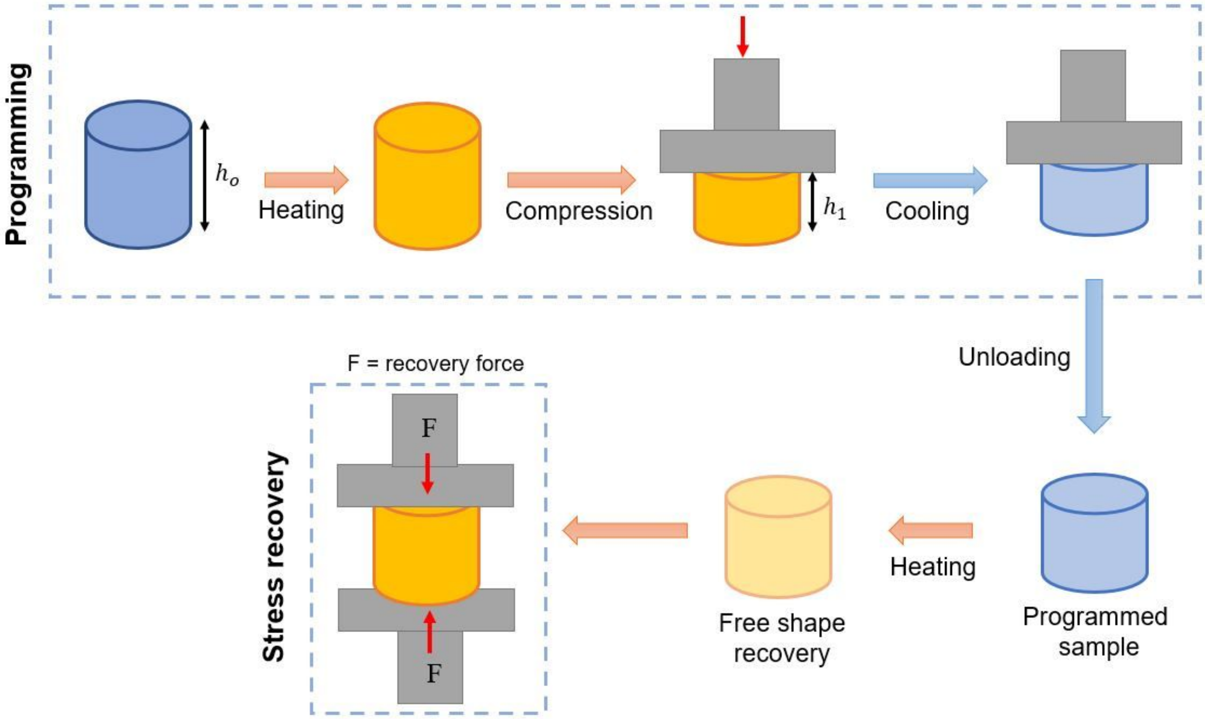

Figure S4. A Schematic diagram of the creation of recovery force during fully constrained shape recovery test

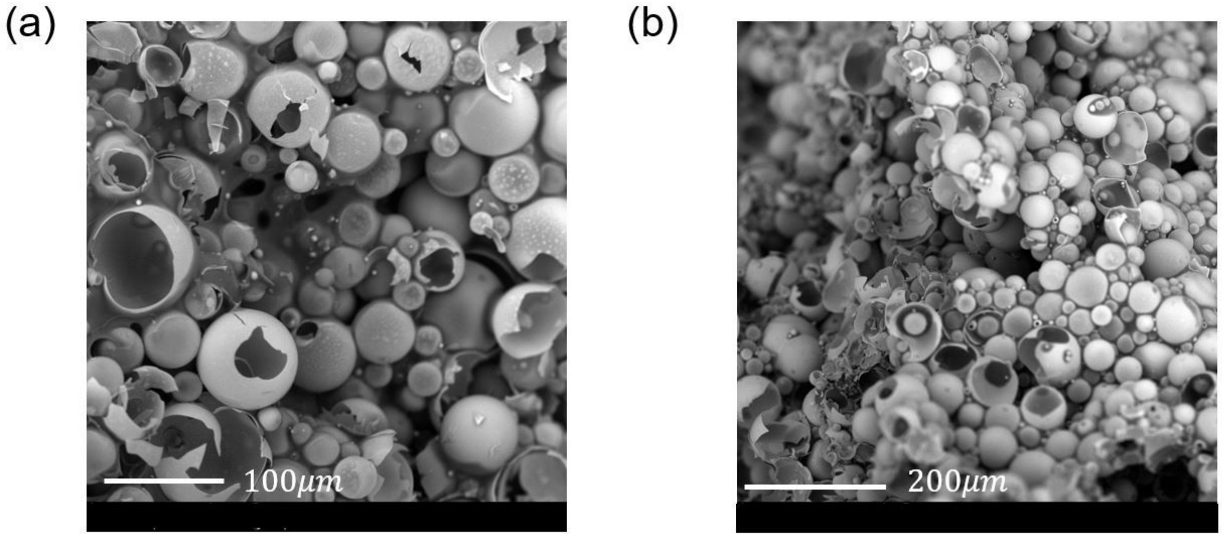

Figure S5. SEM observations of the char residue of the foam containing (a) K1 (b) K15 HGM.

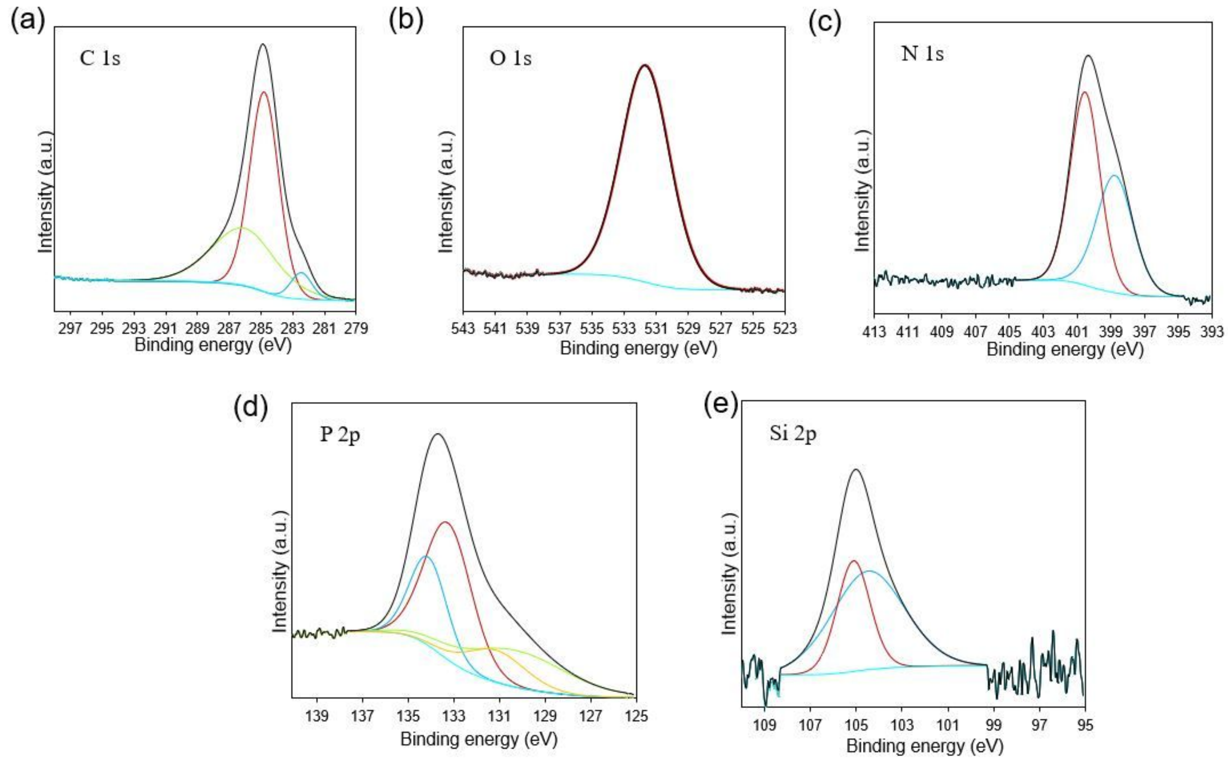

Figure S6. High-resolution (b) C 1s, (c) O 1s, (d) N 1s, (e) P 2p, and (f) Si 2p XPS spectra of the char residue of the syntactic foam containing K1 HGM

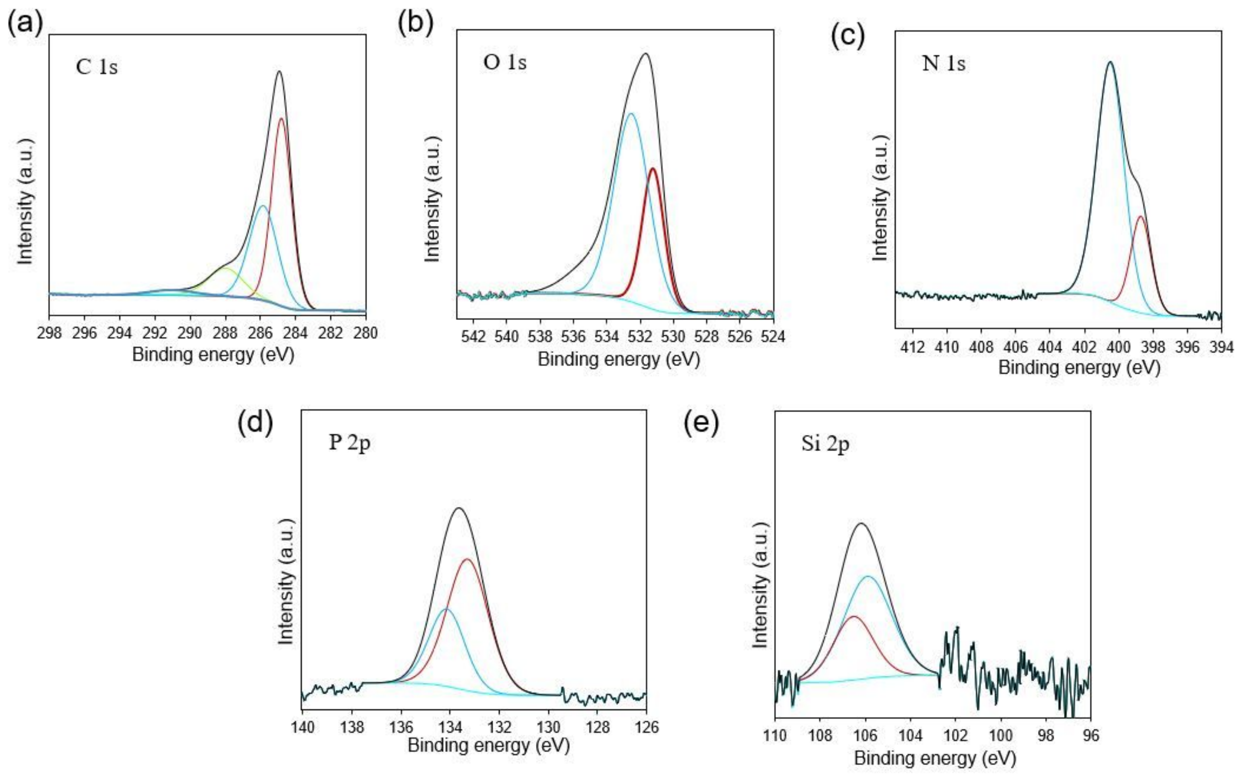

Figure S7. High-resolution (b) C 1s, (c) O 1s, (d) N 1s, (e) P 2p, and (f) Si 2p XPS spectra of the char residue of the syntactic foam containing K15 HGM

Table S1. Density comparison among different syntactic foams.

Sample	Percentage of HGM (%)	Measured Density (g/cc)	Source
K1-SF	40	0.85	Result from this study
K15-SF	40	0.86	Result from this study
K20-SF	40	0.88	Result from this study
Epoxy + K1	40	0.892	[1]
Epoxy + K20	40	0.929	[1]
Epicote 1006 epoxy resin + K15	40	0.72	[2]
PCL + Q-CEL 6014 HGM	40	0.7	[3]
DGEBA based epoxy resin + microballoons	40	0.735-0.846	[4]
diglycidyl ether of bisphenol-A based epoxy + microspheres (S22)	65	0.493	[5]
diglycidyl ether of bisphenol-A based epoxy + microspheres (K46)	65	0.651	[5]
Cycloaliphatic epoxy resin + K15	40	0.70	[6]
Cycloaliphatic epoxy resin +K46	40	0.82	[6]

Table S2. Summary of the thermogravimetric analysis (TGA) of the syntactic foams

Sample	Temperature corresponding to 2% weight loss ($T_{2\%}$ ℃)	Temperature corresponding to maximum weight loss (T_{max} ℃)	Char residue at 500℃ (%)
K1-SF	350	373	26
K15-SF	346	371	32.5
K20-SF	341	365	25.8
HTSMP [7]	405	454	21.6

Table S3. Summary of shape memory characteristic parameters and recovery stress of the syntactic foam (SF) containing different HGMs.

Sample	Fixity ratio (%)	Recovery ratio (%)	Recovery stress (MPa)
K1-SF	56.7	78.85	3.8
K15-SF	57.6	82.06	4.7
K20-SF	62.8	88.46	6.8
HTSMP	58	93.1	35.3 at 20% strain

Reference

1. Zhu, B., et al., Thermal, dielectric and compressive properties of hollow glass microsphere filled epoxy-matrix composites. Journal of Reinforced Plastics and Composites, 2012. 31(19): 1311-1326.
2. Wouterson, E.M., et al., Specific properties and fracture toughness of syntactic foam: Effect of foam microstructures. Composites Science and Technology, 2005. 65: 1840–1850.
3. Lu, L., J. Cao, and G. Li, A polycaprolactone-based syntactic foam with bidirectional reversible actuation. Journal of Applied Polymer Science, 2017. 134(34): 45225.
4. Gupta, N. and R. Nagorny, Tensile Properties of Glass Microballoon-Epoxy Resin Syntactic Foams. Journal of Applied Polymer Science, 2006. 102: 1254 –1261.
5. Gupta, N. and E. Woldesenbet, Hygrothermal studies on syntactic foams and compressive strength determination. Composite Structures, 2003. 61(4): 311-320.
6. Ullas, A.V., D. Kumar, and P.K. Roy, Poly(dimethylsiloxane)-toughened syntactic foams. J. APPL. POLYM. SCI., 2018. 135(8).
7. Feng, X. and G. Li, High-temperature shape memory photopolymer with intrinsic flame retardancy and record-high recovery stress. Applied Materials Today, 2021. 23: 101056.
